# Book Notices for February

**Published:** 1869-02

**Authors:** 


### BOOK NOTICES FOR FEBRUARY.

Out of the Fire. This is the title of a new book just published by the National Temperance Society, of 420 pages, 12mo, written by Miss Mary D. Chellis, author of "The Temperance Doctor," "Deacon Sim's Prayers," etc., etc. The well-earned reputation of the author is fully sustained in this new and valuable addition to our Temperance literature. The evils of the drinking customs of society, and the blessings of sobriety and total abstinence, are strikingly developed in the history of various families in the community. The quiet but hallowed influence of a pure-hearted and devoted cripple is felt by an entire village, and the evils of drunkenness, together with the sure and inevitable tendency of moderate drinking, are graphically depicted. The book illustrates the growth of grace in the heart, and shows that

> "Out of the fires of shame and sin,
> God is able to garner in
> A glorious harvest of souls."

This work should have a wide circulation, and be placed in all our Sunday-school libraries. Price $1.25. J. N. Stearns, Publishing Agent, 172 William Street, N. Y.

THE ATLANTIC MONTHLY FOR FEBRUARY has a wide range of articles mostly by well-known writers, as follows: "Malbone: an Oldport Romance." The second part of a serial story, by T. W. Higginson. "The Doorstep." A poem by Edmund C. Stedman. "Our Postal Deficiencies." By E. Hasket Derby. "Co-operative Housekeeping." The fourth of a series of papers that should be read by all housekeepers. "Charles Baudelaire, Poet of the Malign." A sketch of the character and writings of this noted French poet, by Eugene Benson. "Consumption in America." The second paper of a very valuable series on a subject of the greatest practical importance, by Henry I. Bowditch, M.D. "The Bee and the Rose." A Poem. "Ritualism in England." By Arthur Pember. "Proud Music of the Sea-Storm." A poem, by Walt Whitman. "The New Education." A general statement of the demand of the times for a more practical system of education, and an account of the Scientific and Technological Schools founded to furnish it. By Charles W. Eliot. "Birth of the Solar System." By James D. Whelpley. "Love in Mount Lebanon." By J. W. DeForest. "Tribute of a Loving Friend to the Memory of a Noble Woman." (The Duchess of Sutherland.) By Mrs. H. B. Stowe. "Our Four Servants." "Reviews and Literary Notices."

Fields, Osgood & Co., Publishers, Boston. (Successors to Ticknor & Fields.)

AVOIDING SICKNESS. The house of Hurd & Houghton, 459 Broome Street, New York, have just published a very handsome 12mo of 366 pages, entitled, "How not to be Sick," being a sequel to the "Philosophy of Eating," by Albert J. Bellows, M.D., late Professor of Chemistry, Physiology and Hygiene, etc. The volume has attracted considerable attention, and liberal extracts have been made from it in the papers. The book is in the interest of homeopathy, but will command the attention and meet with the approbation of intelligent men of all the schools, because founded on observed facts. Without commending all that the industrious author has said, attention is drawn to some of the topics discussed that the reader may see for himself what a mine of practical information is opened for his thoughtful attention: Food for thinking men, ditto for laboring men, for sedentary men, for winter, for summer, for dyspeptics, for chronic disease; is animal food injurious? corpulence and leanness prevented by diet; ditto gout, etc., etc. We will send a volume, post-paid, to any one who sends us three subscriptions to Hall's Journal of Health, 176 Broadway, New York, being four dollars and a half.

J. S. Redfield, 140 Fulton Street, New York, has issued another edition of "Modern Women and What is said of Them." Sent for $2, free of postage, by addressing him as above. The introduction, by Mrs. Lucie Gilbert Calhoun, is an admirable production, doing credit to her head and heart.

Readers of The Tribune who have been familiar with its editorial columns for the last eight or ten years, cannot have failed to remark that every few days there has appeared an article of striking ability in the vein of satirical criticism. These articles have not only the merit of being well written, but they have been opportune criticisms of persons and events, over which thousands of our readers have grown merry, and there are few of them, we imagine, who will not be glad to know that a collection of these contributions has been made by the author, Mr. C. T. Congdon, and that the same is about to be published in book form by J. S. Redfield, with an introduction by Mr. Greeley.

The Astor House, New York, is very near our office, and those who come to the city to see us, would do well to make it their home; it has been recently put in complete order, and is conducted by the Messrs. Stetson in a manner not surpassed in any first class hotel on this continent for convenience, comfort and enjoyment.

